# A Novel Mouse Model of Enteric Vibrio parahaemolyticus Infection Reveals that the Type III Secretion System 2 Effector VopC Plays a Key Role in Tissue Invasion and Gastroenteritis

**DOI:** 10.1128/mBio.02608-19

**Published:** 2019-12-17

**Authors:** Hyungjun Yang, Marcela de Souza Santos, Julia Lee, Hong T. Law, Suneeta Chimalapati, Elena F. Verdu, Kim Orth, Bruce A. Vallance

**Affiliations:** a Division of Gastroenterology, Hepatology and Nutrition, BC Children’s Hospital Research Institute and the University of British Columbia, Vancouver, Canada; b Department of Molecular Biology, University of Texas Southwestern Medical Center, Dallas, Texas, USA; c Farncombe Family Digestive Health Research Institute, McMaster University, Hamilton, Ontario, Canada; d Howard Hughes Medical Institute, University of Texas Southwestern Medical Center, Dallas, Texas, USA; e Department of Biochemistry, University of Texas Southwestern Medical Center, Dallas, Texas, USA; UC Berkeley

**Keywords:** T3SS, *Vibrio parahaemolyticus*, gastroenteritis, *in vivo* model, pathogenesis

## Abstract

The Gram-negative marine bacterium Vibrio parahaemolyticus is a common cause of infectious gastroenteritis due to the ingestion of contaminated seafood. Most virulent V. parahaemolyticus strains encode two type III secretion systems (T3SS1 and T3SS2); however, the roles they and their translocated effectors play in causing intestinal disease remain unclear. While studies have identified T3SS1 effectors as responsible for killing epithelial cells in culture, the T3SS2 effectors caused massive epithelial cell disruption in a rabbit ileal loop model. Additional models are thus needed to clarify the pathogen-host interactions that drive V. parahaemolyticus-associated gastroenteritis. Germfree mice were infected with a pathogenic clinical isolate of V. parahaemolyticus, RIMD2210633 (RIMD). The pathogen was found to adhere to as well as invade the cecal mucosa, accompanied by severe inflammation and dramatic mucosal damage, including widespread sloughing of infected epithelial cells. Mice infected with a V. parahaemolyticus strain lacking the T3SS1 (POR2) also developed severe pathology, similar to that seen with RIMD. In contrast, the *ΔT3SS2* strain (POR3) appeared unable to invade the intestinal mucosa or cause any mucosal pathology. Confirming a role for TS332 effectors, a strain expressing the T3SS2 but lacking VopC (POR2*ΔvopC*), a T3SS2 effector implicated in epithelial cell invasion in culture, was strongly attenuated in invading the intestinal mucosa and in causing gastroenteritis, although infection with this mutant resulted in more pathology than the *ΔT3SS2* strain. We thus present an experimental system that enables further characterization of T3SS effectors as well as the corresponding host inflammatory response involved in the gastroenteritis caused by invasive V. parahaemolyticus.

## Abstract (precis)

Vibrio parahaemolyticus causes severe gastroenteritis following consumption of contaminated seafood. Global warming has allowed this pathogen to spread worldwide, contributing to recent outbreaks. Clinical isolates are known to harbor an array of virulence factors, including T3SS1 and T3SS2; however, the precise role these systems play in intestinal disease remains unclear. There is an urgent need to improve our understanding of how V. parahaemolyticus infects hosts and causes disease. We present a novel mouse model for this facultative intracellular pathogen and observe that the T3SS2 is essential to pathogenicity. Moreover, we show that the T3SS2 effector VopC, previously shown to be a Rac and Cdc42 deamidase that facilitates bacterial uptake by nonphagocytic cells, also plays a key role in the ability of V. parahaemolyticus to invade the intestinal mucosa and cause gastroenteritis. This experimental model thus provides a valuable tool for future elucidation of virulence mechanisms used by this facultative intracellular pathogen during *in vivo* infection.

## INTRODUCTION

Vibrio parahaemolyticus is a Gram-negative bacterium found in warm marine environments throughout the world (1–3). It is the leading cause of acute gastroenteritis associated with the consumption of undercooked seafood (1, 3). Disease symptoms include diarrhea, vomiting, abdominal cramping, and low-grade fever, which typically resolve within 2 to 3 days; however, infection can escalate to a potentially lethal septicemia in immunocompromised individuals (1, 3). To date, clinical isolates of V. parahaemolyticus such as RIMD2210633 (RIMD) have been found to encode a number of virulence factors, including two hemolysins (thermostable direct hemolysins [TDHs]), two type III secretion systems (T3SS1 and T3SS2), two type VI secretion systems (T6SS1 and T6SS2), and numerous adhesins, including MAM7 and MSHA (3–7).

Several animal models have been developed to study the enterotoxicity caused during V. parahaemolyticus infection, including bacterial injections into rabbit ligated ileal loops and orogastric inoculation of piglets and infant rabbits (8–11). Altogether, these models recapitulate many of the histopathological manifestations observed in the intestines of infected human patients, such as intestinal epithelial cell (IEC) denudation, mucosal and submucosal edema, lamina propria congestion, and infiltration of inflammatory cells (12). Importantly, all these models identified the V. parahaemolyticus T3SS2 as the principal virulence factor for disease development (8–10). Even so, despite its being a facultative intracellular pathogen, it remains unclear how V. parahaemolyticus interacts with the intestinal epithelium of animal models and whether it invades mucosal tissues (13, 14). Interestingly, similar uncertainties were an issue with the facultative intracellular pathogen Salmonella enterica serovar Typhimurium, until researchers began analyzing the ceca of infected mice within 24 h postinfection (p.i.) (15).

T3SSs are secretory apparatuses used by many Gram-negative pathogens to deliver specialized virulence proteins, termed effectors, into infected host cells (16). Effectors often mimic the structure and function of eukaryotic proteins and thus hijack critical cellular machinery, such as the actin cytoskeleton, cargo trafficking, and the innate immune response. Through these actions, T3SS-dependent effectors promote successful and prolonged bacterial infections (17, 18). Whole-genome sequencing of V. parahaemolyticus RIMD revealed the presence of two clusters of genes encoding the T3SS. To date, eight effectors have been found to be delivered by V. parahaemolyticus’ T3SS2: VopA is an acetyltransferase that inhibits mitogen-activated protein (MAP) kinase (MAPK) MKKs (19, 20), VopT is an ADP ribosyltransferase targeting the Rho GTPase Ras (21), VopO binds GEF-H1 (22), VopV is an actin-bundling protein (23, 24), VopZ inhibits TAK-1 to suppress the MAPK and NF-κB pathways (25), VopL nucleates actin filaments resulting in suppression of reactive oxygen species (26–30), and VPA1380’s activity remains elusive (31). An eighth effector, VopC, deamidates and activates the Rho GTPases Cdc42 and Rac-1 (32, 33), resulting in actin polymerization and lamellipodium formation at sites of bacterial attachment to host cells, leading to bacterial internalization (32, 33). Notably, the majority of these effector functions have only been assessed in cell culture. Defining how T3SS effectors function *in vivo* to promote V. parahaemolyticus infection, as well as their effects on the host inflammatory response, will be critical in the search for novel approaches to prevent these infections.

Here, we report the development of a novel animal model for V. parahaemolyticus-driven gastroenteritis. Since the resident gut microbiota inhibited this pathogen’s ability to colonize the mouse intestine, we orogastrically infected germfree mice with the clinical V. parahaemolyticus isolate RIMD. Infected mice carried heavy pathogen burdens, with infection and tissue damage focused on the cecum. Immunostaining revealed the cecal mucosa to be heavily invaded by RIMD, with infection leading to widespread IEC sloughing and neutrophil infiltration. Both histological features are observed in the intestines of patients suffering V. parahaemolyticus infection and in previously developed animal models, validating this model (10–12, 34). Using deletion strains derived from RIMD, our studies confirmed that enterotoxicity is largely dependent on the T3SS2 and, importantly, revealed that the T3SS2 effector VopC contributes to pathogen virulence *in vivo*. Specifically, mucosal invasion by the bacterium, as well as tissue pathology, was greatly attenuated in the absence of this effector. We believe this model will aid future studies in dissecting the individual contributions of other T3SS2 effectors toward V. parahaemolyticus’ enterotoxicity, while the array of tools suitable for studying the murine immune system will help clarify how the host responds to and defends against this invasive pathogen.

## RESULTS

### V. parahaemolyticus is unable to colonize the intestines of SPF mice.

To evaluate the ability of V. parahaemolyticus to colonize the intestines of mice in the presence of the resident gut microbiota, specific-pathogen-free (SPF) C57BL/6 mice were gavaged with 10^9^ CFU of the V. parahaemolyticus clinical isolate RIMD. Following infection, their stool was collected at 4 h postinfection (p.i.), and the mice were subsequently euthanized at 21 h p.i. for further sample collection. While V. parahaemolyticus was readily detectable in the feces at 4 h p.i. (∼10^6^ CFU/g) (see Fig. S1A in the supplemental material), the pathogen was cleared from the gut by 21 h. Similarly, no culturable V. parahaemolyticus was recovered from the ceca or colons (Fig. S1B) of infected mice at this time point, nor was any macroscopic or microscopic pathology noted (Fig. S1C and D).

10.1128/mBio.02608-19.1 FIG S1 Streptomycin pretreatment reduces intestinal colonization resistance against V. parahaemolyticus RIMD2210633 in SPF mice. SPF C57BL/6 mice were pretreated with PBS or streptomycin by oral gavage followed by oral infection with V. parahaemolyticus on the next day. (A and B) Colonization of V. parahaemolyticus was determined from the feces collected at 4 hours and 21 hours (A) and from cecal and colonic tissues at 21 hours (B) after infection. (C) Gross pathology of cecum and colon from nontreated/streptomycin-treated/no-antibiotic plus V. parahaemolyticus-infected and streptomycin plus V. parahaemolyticus-infected SPF C57BL/6 mice. (D) Representative images of ceca and colons stained with H&E stain from 21 hours p.i. indicated no significant histological damage in infected SPF mice. (E) Representative image showing colonization of V. parahaemolyticus in the cecum. V. parahaemolyticus is in green, actin in red, and nucleus in blue. In the graphs, bars show the median, and each symbol represents an individual mouse from 2 to 3 independent experiments. **, *P* < 0.01; ***, *P* < 0.001, by Mann-Whitney test. nd, not detected. Download FIG S1, PDF file, 0.6 MB. Copyright © 2019 Yang et al. 2019 Yang et al. This content is distributed under the terms of the Creative Commons Attribution 4.0 International license.

### Streptomycin pretreatment reduces colonization resistance to V. parahaemolyticus.

Based on the inability of V. parahaemolyticus to effectively colonize the intestines of mice carrying a normal gut microbiota, we next tested whether antibiotic-based depletion of commensal microbes would facilitate pathogen colonization. Mice were gavaged with streptomycin (20 mg) and 24 h later orally infected with 10^9^ CFU of RIMD. Compared to untreated mice, fecal shedding was modestly higher in the streptomycin-treated mice at 4 h p.i. (∼10^8^ CFU/g of feces) and remained similarly high at 21 h p.i. (Fig. S1A). Moreover, unlike untreated mice, ∼10^6^ CFU/g of V. Parahaemolyticus was recovered from the cecal and colonic tissues of infected, streptomycin-pretreated mice at this time point (Fig. S1B). Even so, little if any macroscopic or microscopic intestinal pathology was noted (beyond the enlarged ceca caused by streptomycin treatment) in the infected mice (Fig. S1C and D). Correspondingly, immunofluorescence staining revealed V. parahaemolyticus present in the cecal lumen as well as potentially adherent to the intestinal epithelium, but not invading gut tissues (Fig. S1E). These results confirm that microbiota-based colonization resistance (35) prevents V. parahaemolyticus from establishing residence in the intestines of conventionally raised mice (as previously shown [36]).

### V. parahaemolyticus heavily colonizes the intestines of germfree mice.

**FIG 1:**
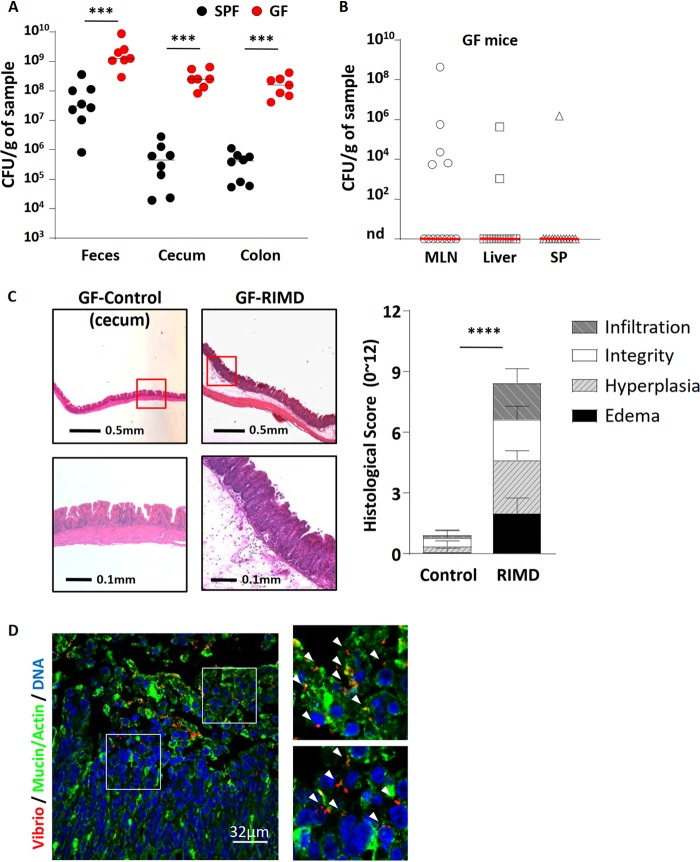
V. parahaemolyticus RIMD2210633 heavily colonizes the intestines and causes cecitis in germfree (GF) mice. V. parahaemolyticus was orally gavaged into germfree C57BL/6 mice that were euthanized at 21 h p.i. for analysis. (A) Colonization (CFU) of V. parahaemolyticus was measured from feces and cecal and colonic tissue and compared to streptomycin-pretreated SPF mice that were infected with V. parahaemolyticus. (B) V. parahaemolyticus RIMD rarely translocated into systemic tissues: mesenteric lymph nodes (MLN), liver, and spleen (SP). nd, not detected. (C) Representative H&E staining images and histologic scores of infected and uninfected ceca of germfree mice. (D) Representative immunostaining images of cecum show V. parahaemolyticus RIMD (red; noted by white arrowheads) invasion into cecal tissues. Mucin and actin are shown in green, and DNA is shown in blue. In the graphs, bars show the median (A and B) or the mean ± SEM (C), and each symbol represents an individual mouse from two independent experiments. ***, *P* < 0.001, and ***, *P* < 0.0001, by Mann-Whitney test (A and B) or Student’s *t* test (C).

Based on the increased susceptibility to V. parahaemolyticus infection exhibited by antibiotic-treated mice, we hypothesized that the remaining commensal microbes that survived streptomycin treatment (as previously described [37–39]) might still be preventing the virulence of V. parahaemolyticus. To test this, we used germfree C57BL/6 mice, gavaging them with 10^9^ CFU of the clinical isolate RIMD. Mice were euthanized at different time points (12, 18, 21, and 24 h) postinfection, and fecal samples were collected to measure intestinal colonization. The highest and most consistent intestinal burdens were observed at 21 h p.i. (Fig. S2A), leading us to focus on this time point. Notably, we recovered significantly higher numbers (∼1 × 10^8^ to 5 × 10^9^ CFU) of V. parahaemolyticus from the feces, as well as cecal and colonic tissues of germfree mice, compared to streptomycin-pretreated SPF mice (Fig. 1A). In contrast, we noted little if any pathogen colonization of the small intestine (not shown). These results confirm that microbiota-based colonization resistance plays a key role in preventing V. parahaemolyticus from optimally colonizing the intestines of conventionally raised mice.

10.1128/mBio.02608-19.2 FIG S2 Time course of colonization, as well as macroscopic and histologic appearances of large bowel during infection. (A) Pathogen shedding (CFU) in feces collected at 12, 18, 21, and 24 hours aftert V. parahaemolyticus RIMD infection in germfree mice. Bars show the median from two pooled independent experiments. (B) Representative macroscopic images of uninfected and infected large bowel. (C) Representative H&E staining image of colonic cross sections from uninfected and infected germfree mice. Download FIG S2, PDF file, 0.4 MB. Copyright © 2019 Yang et al. 2019 Yang et al. This content is distributed under the terms of the Creative Commons Attribution 4.0 International license.

We also examined the mesenteric lymph nodes (MLN), liver, and spleen of infected mice for the presence of V. parahaemolyticus. Finding high pathogen numbers at systemic tissue sites could indicate that the intestinal barriers within germfree mice are too immature to properly contain the infection. In contrast, V. parahaemolyticus was only rarely recovered from these systemic sites (Fig. 1B), suggesting the intestinal barriers of the infected germfree mice were sufficiently mature to contain V. parahaemolyticus within the gastrointestinal (GI) tract, preventing large-scale translocation to systemic tissues.

### V. parahaemolyticus causes severe gastroenteritis in the ceca of germfree mice.

Infection with the RIMD strain led to a reduction in cecal size as well as softer stool in germfree mice (Fig. S2B). Histology revealed severe intestinal pathology including a thickening of the cecal mucosa, along with IEC sloughing, transmural inflammatory cell infiltration, crypt abscesses, and dramatic submucosal edema (Fig. 1C). Tissue scoring performed in a blind manner confirmed the severe intestinal pathology seen in this model (Fig. 1C). While pathology was focused in the cecum, modest pathology was also observed in the colon (Fig. S2C), whereas the small intestine appeared comparatively unaffected by infection (not shown).

### V. parahaemolyticus invades the cecal mucosa of germfree mice.

To better define the basis for the severe gastroenteritis seen in infected germfree mice, the location of the RIMD strain within the ceca of infected mice was assessed using immunofluorescence. Similar to the staining seen in streptomycin-pretreated mice (Fig. S1E), numerous V. parahaemolyticus bacteria were present within the cecal lumen, and even larger numbers of V. parahaemolyticus were also found adherent to the mucosal surface (Fig. 1D). These foci of bacteria were patchy and often present near areas of overt IEC sloughing. Moreover, V. parahaemolyticus was found widely distributed throughout the cecal mucosa, either individually or in small clusters indicating that it had invaded the cecal mucosal tissue (Fig. 1D). Thus, in this model, although many V. parahaemolyticus bacteria remain extracellular, and adherent to the cecal epithelial surface, there is also overt and widespread invasion of the cecal mucosa by the RIMD strain of V. parahaemolyticus.

### Infection triggers increased intestinal inflammation.

**FIG 2:**
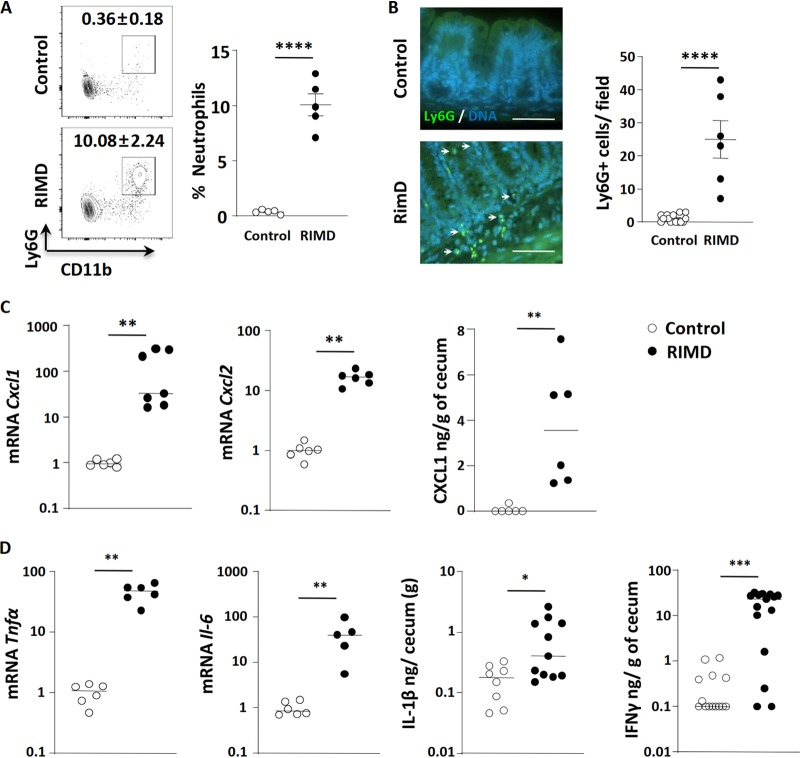
V. parahaemolyticus RIMD2210633 infection triggers increased intestinal inflammation. V. parahaemolyticus-infected germfree C57BL/6 mice were euthanized at 21 h p.i. for analysis. (A) Flow cytometry analysis showed significant CD11b^+^ Ly6G^+^ neutrophil infiltration into the lower gastrointestinal tract (cecum and colon). (B) Immunostaining showed Ly6G^+^ neutrophil (green; shown by white arrows) infiltration into the cecal mucosa and submucosa during V. parahaemolyticus infection. DNA in cell nuclei were stained in blue. Bars, 50 μm. (C) Transcription of neutrophil-attracting chemokine genes *Cxcl1* and *Cxcl2* increased in infected cecal tissues, as did the protein level of CXCL1 as determined by ELISA from cecal homogenates. (D) Increased expression/production of proinflammatory cytokines (*Tnfα* and *Il-6* by qPCR and IL-1β and IFN-γ by ELISA) in the infected cecum. In the graphs, bars show the mean ± SEM (A and B) or the median (C and D), and each symbol represents an individual mouse from 2 to 3 independent experiments. *, *P* < 0.05; **, *P* < 0.01; ***, *P* < 0.001; ****, *P* < 0.0001, by two-tailed Student’s *t* test (A and B) or Mann-Whitney test (C and D).

Continuing our histological analysis, hematoxylin-and-eosin (H&E)-stained slides revealed a dramatic influx of neutrophils into the cecal mucosa and submucosa of infected mice (Fig. 1C). To confirm this, as well as better define the host response, flow cytometry analysis and immunofluorescent staining for neutrophils were performed. As expected, under baseline conditions, there were few if any neutrophils (Ly6G positive) present within the cecal or colonic tissues of germfree mice (Fig. 2A and B). Upon infection, there was a dramatic influx of neutrophils into these tissues (Fig. 2A), with immunofluorescent staining revealing that the majority of Ly6G-positive neutrophils were located within the mucosal and submucosal tissues (Fig. 2B).

Such inflammatory cell recruitment typically occurs in response to the increased expression of chemokines. Compared to uninfected cecal tissues, infection led to the significant upregulation (10- to 500-fold) in transcription of the neutrophil-attracting chemokines *Cxcl1* and *Cxcl2* (Fig. 2C). We also assessed protein levels for CXCL1 (KC), and they were also increased (Fig. 2C). In addition, we examined whether infection led to the induction of proinflammatory cytokines, finding significantly increased transcription of the *Tnf-α* and *Il-6* genes (Fig. 2D) as well as significantly elevated protein levels for IL-1β and IFN-γ as determined by ELISA of cecal protein lysates (Fig. 2D). Thus, V. parahaemolyticus induces a strong inflammatory response in the infected mucosal tissues of germfree mice.

### Infection leads to dramatic increases in epithelial cell death and proliferation.

**FIG 3:**
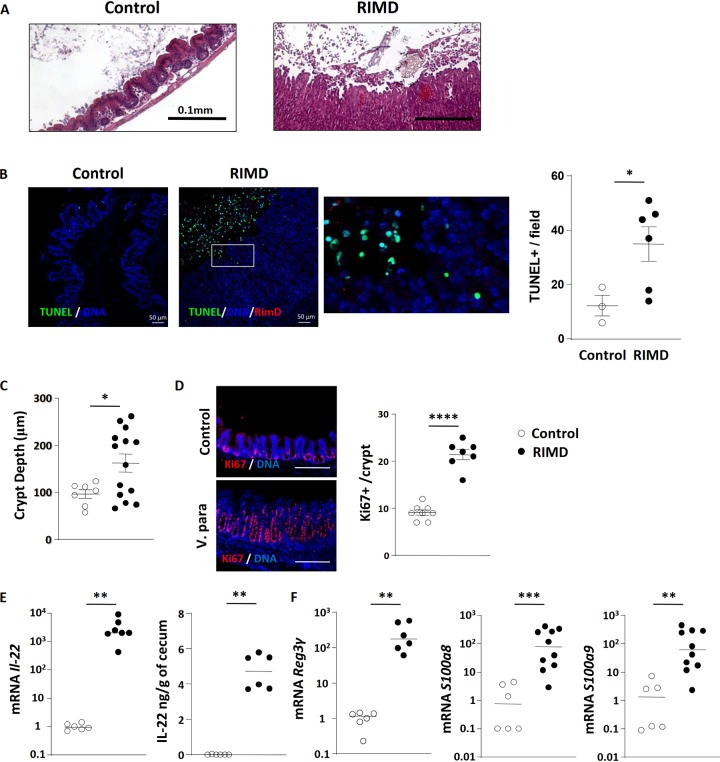
Infection leads to dramatic increases in epithelial cell death and proliferation. (A) Representative H&E-stained images of control and infected ceca showed significant epithelial sloughing into the lumen during infection. (B) Representative image of TUNEL staining and quantification of TUNEL-positive cells. Cecal tissues from control and RIMD-infected mice were stained for DNA (blue), TUNEL (green), and V. parahaemolyticus (red). Bars, 50 μm. TUNEL-positive cells/high-power field were also quantified and enumerated. (C) Measurement of crypt depths in control and RIMD-infected ceca. (D) Ki-67 staining (red) is used to demonstrate increased epithelial proliferation in RIMD-infected ceca. The nuclei were stained with DAPI (blue). Bars, 200 μm. (E) Increased expression of IL-22 at the mRNA and protein level in infected ceca. (F) Increased antimicrobial peptide (*Reg3γ*, *S100a8*, and *S100a9*) gene transcription in infected ceca. In the graphs, bars show the mean ± SEM (B to D) or the median (E and F), and each symbol represents an individual mouse from 2 to 3 independent experiments. *, *P* < 0.05; **, *P* < 0.01; ***, *P* < 0.001; ****, *P* < 0.0001, by two-tailed Student’s *t* test (B to D) or Mann-Whitney test (E and F).

Aside from inflammatory cell infiltration, other infection-induced pathologies included the widespread sloughing of IECs from the surface of cecal crypts (Fig. 3A). To address whether the IEC sloughing reflected increased apoptosis, TUNEL (terminal deoxynucleotidyltransferase-mediated dUTP-biotin nick end labeling) staining was performed. As expected, the majority of the sloughed IECs found in the cecal lumen were TUNEL positive, indicating they had undergone apoptosis. When we examined IECs on the mucosal surface that were in the process of sloughing, only a small fraction was TUNEL positive (Fig. 3B), and there was no correlation between IECs infected by V. parahaemolyticus and the apoptotic marker. This suggests that while infection leads to IEC sloughing into the cecal lumen, the cells undergo apoptosis after they have sloughed, rather than in direct response to infection. This agrees with findings made using the rabbit ileal model of V. parahaemolyticus infection (10).

We also noted a thickened cecal mucosa during infection, so we measured crypt depths and confirmed they significantly increased following infection (Fig. 3C). To examine if this reflected an increase in IEC proliferation, tissues were immunofluorescently stained for a proliferation marker (Ki-67) and host cell nuclei (4′,6-diamidino-2-phenylindole [DAPI]). As shown in Fig. 3D, enumeration revealed a striking doubling in Ki-67-positive (proliferating) IECs/crypt during infection. Thus, despite the widespread sloughing of IECs, the concomitant increase in IEC proliferation not only replaces those lost cells but also leads to overt crypt hyperplasia. While there are many growth factors that can increase IEC proliferation, we and others have noted that the cytokine interleukin-22 (IL-22) frequently drives this pathological process during mouse models of infectious gastroenteritis (40–42). As expected, *Il-22* mRNA levels underwent a dramatic increase during infection (>1,000-fold), while IL-22 protein levels also increased (Fig. 3E). Aside from inducing IEC proliferation, IL-22 can also promote the expression of antimicrobial factors such as the lectin Reg3γ. Correspondingly, transcription of *Reg3*γ as well as the genes encoding the antimicrobial factors *S100a8* and *S100a9* was also strongly induced (100-fold) during infection, potentially to promote host defense (Fig. 3E).

### The T3SS2 is required for V. parahaemolyticus colonization and gastroenteritis.

**FIG 4:**
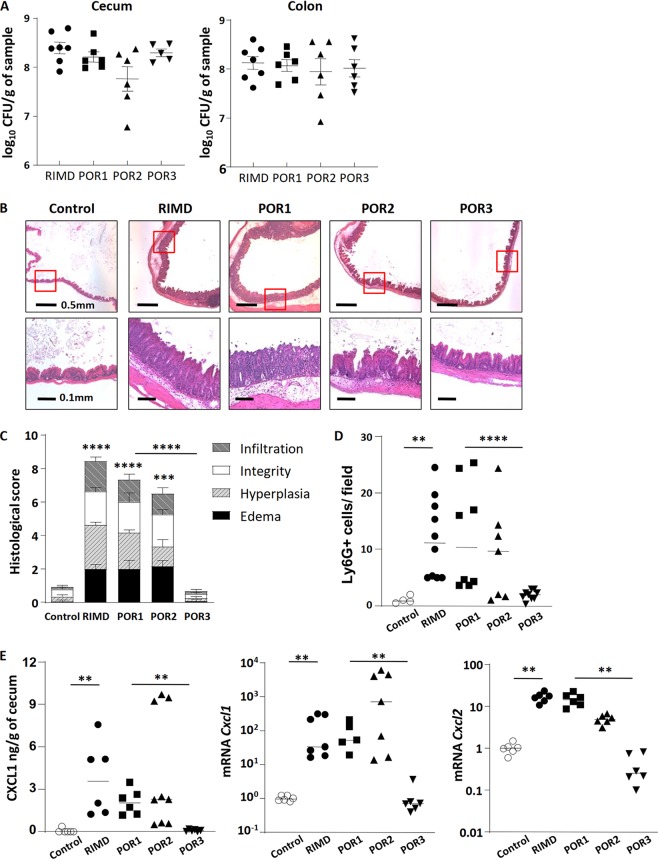
The T3SS2 is required for V. parahaemolyticus RIMD2210633-induced gastroenteritis. (A) Germfree C57BL/6 mice were infected with RIMD2210633 or the POR1, POR2, and POR3 mutant strains and euthanized at 21 h p.i. for analysis. Bacterial burdens (CFU) in the cecal and colonic tissues were comparable between wild-type (WT) and mutant strains. (B) Representative H&E-stained images of ceca. Lower images are zoomed-in pictures of the indicated area (red box) of the upper images. (C) Histological scores of cecal pathology. (D) Ly6G^+^ neutrophil infiltration was quantified by immunostaining and enumerating positive cells/high-power field. (E) Neutrophil chemoattractant genes *Cxcl1* and *Cxcl2* were determined by ELISA and qPCR. In the graphs, bars show the mean ± SEM (C) or the median (D and E), and each symbol represents an individual mouse from 2 to 3 independent experiments. **, *P* < 0.01; ***, *P* < 0.001; ****, *P* < 0.0001, by Student’s *t* test (C) or Mann-Whitney test (D and E).

To take further advantage of this robust model of V. parahaemolyticus-induced gastroenteritis, we next tested whether the TDHs, T3SS1, or T3SS2 impacted the ability of V. parahaemolyticus to colonize germfree mice and cause gastroenteritis. To do so, several mutant strains derived from clinical isolate RIMD, referred to collectively as the POR strains and individually as POR1, POR2, and POR3 (Table S1), were tested (34). Germfree mice were infected for 21 h, and all 3 mutant strains were recovered from the cecum and colon at levels (∼10^8^ CFU/ml) roughly similar to the recovery of RIMD (Fig. 4A). As others have previously shown (43), luminal colonization of the GI tract of germfree mice by pathogenic (and commensal) bacteria occurs very quickly and does not require specific virulence factors (44–46), in this case the TDH, T3SS1, or T3SS2 of V. parahaemolyticus.

10.1128/mBio.02608-19.5 TABLE S1 V. parahaemolyticus and its mutant strains. Download Table S1, PDF file, 0.4 MB. Copyright © 2019 Yang et al. 2019 Yang et al. This content is distributed under the terms of the Creative Commons Attribution 4.0 International license.

In contrast to their limited impact on colonization, these factors did contribute to the resulting intestinal pathology. Infection with POR1, which lacks TDHs but contains both a functional T3SS1 and T3SS2, caused a pathology phenotype/score similar to that caused by RIMD. Crypt abscesses and hyperplasia, submucosal edema, and inflammatory cell infiltration were all observed at 21 h p.i. (Fig. 4B and C). In mice infected with POR2, which lacks both TDHs and a functional T3SS1 but does possess a functional T3SS2, the inflammatory phenotype and pathology score were modestly but not significantly reduced compared to that seen with POR1 or with RIMD (Fig. 4B and C). In contrast, infection with POR3, which lacks TDHs and a functional T3SS2 but possesses a functional T3SS1, resulted in a severely attenuated inflammatory phenotype. While crypts from the ceca of POR3-infected mice appeared slightly elongated at 21 h p.i. (Fig. 4B), these and other pathology readouts were not significantly different from the phosphate-buffered saline (PBS) control (Fig. 1C and Fig. 4B). Taken together, these data demonstrate that the infectious gastroenteritis in this model occurs independently of the TDHs, and while the T3SS1 may play a modest role in promoting the gastroenteritis, it is the T3SS2 that is critical for the *in vivo* virulence of this pathogen.

We next assessed specific aspects of the inflammatory response, confirming no overt differences in neutrophil infiltrates and chemokine expression between mice infected with the RIMD strain and those infected with the TDH-deficient POR1 strain (Fig. 4D and E). In the POR2-infected mice, significant neutrophil infiltration into the cecal mucosa and submucosa was also observed (Fig. 4D) whereas the ceca of POR3-infected mice appeared normal, and few if any neutrophils were detected in their cecal tissues. Similar to the levels of neutrophil infiltration, protein levels of the neutrophil-attracting chemokine CXCL1, as well as transcription of the *Cxcl1* and *Cxcl2* genes strongly increased in mice infected with the POR1 and POR2 strains but not in the ceca of POR3-infected mice (Fig. 4E). Additional analysis (Fig. S3) of several cytokines (*Tnf-α*, *Il-6*, IL-1β, and IFN-γ) and antimicrobial factors (*Reg3γ*, *S100a8*, and *S100a9*) found to increase during RIMD infection showed a similar trend, i.e., they were also strongly induced by infection with POR1 or POR2 but not POR3.

10.1128/mBio.02608-19.3 FIG S3 Proinflammatory cytokine and antimicrobial factor production in the ceca of mice infected with different strains of V. parahaemolyticus. (A) mRNA levels of proinflammatory cytokines (*Tnfα* and *Il-6*) were measured by qPCR of cecal tissues. (B) IL-1β and IFN-γ protein levels were quantified by ELISA from cecal homogenates of mice infected with RIMD or POR mutant strains. (C) Antimicrobial factor expression was determined by qPCR from the ceca of mice infected with RIMD or POR mutant strains 21 hours p.i. In the graphs, bars show the median (A and C) or mean ± SEM (B), and each symbol represents an individual mouse from 3 independent experiments. *, *P* < 0.05; **, *P* < 0.01; ***, *P* < 0.001, by Mann-Whitney test (A and C) or two-tailed Student’s *t* test (B). Download FIG S3, PDF file, 0.8 MB. Copyright © 2019 Yang et al. 2019 Yang et al. This content is distributed under the terms of the Creative Commons Attribution 4.0 International license.

**FIG 5:**
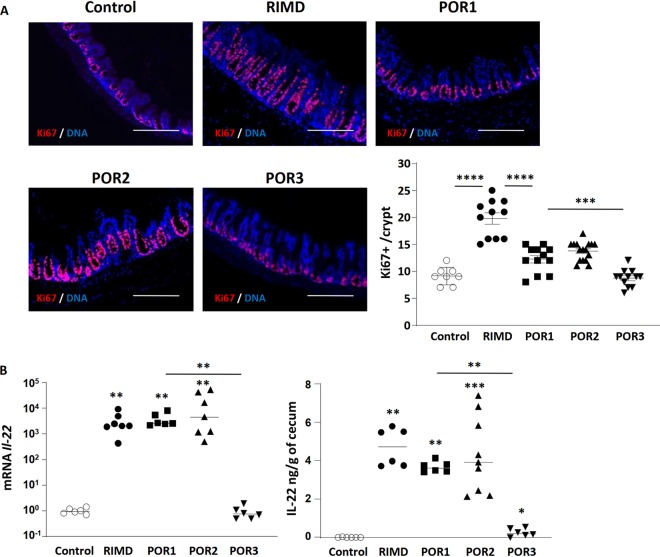
T3SS2 is required for infection-induced increases in IL-22 and epithelial cell proliferation. (A) Ki-67 staining (red) shows that the increase in IEC proliferation seen in cecal tissues infected with wild type (WT) (RIMD2210633) is reduced in tissues infected by the POR1 or POR2 strain and abrogated in POR3-infected ceca. The nuclei were stained with DAPI (blue). Bars, 200 μm. (B) IL-22 production (mRNA and protein) was increased in cecal tissues infected by RIMD, POR1, and POR2 strains but not after POR3 infection. In the graphs, bars show the mean ± SEM (A) or the median (B), and each symbol represents an individual mouse from 2 to 3 independent experiments. *, *P* < 0.05; **, *P* < 0.01; ***, *P* < 0.001; ****, *P* < 0.0001, by two-tailed Student’s *t* test (A) or Mann-Whitney test (B).

We next assessed IEC proliferation in mice infected with the different strains of V. parahaemolyticus. As noted above, in response to infection with RIMD, the cecal epithelium undergoes increased proliferation as measured by Ki-67-positive staining. While this proliferative response was also seen in mice infected with the POR1 and POR2 strains, their increases in Ki-67-positive proliferation over baseline were not as high as those seen with the RIMD strain (Fig. 5A), suggesting subtle differences in their intestinal pathology. As expected, Ki-67-positive staining and localization in the ceca of POR3-infected mice (Fig. 5A) appeared similar to the PBS control. Along with the IEC proliferation data, *Il-22* mRNA and protein levels in the infected ceca were also measured. In keeping with the proliferation data, *Il-22* transcription and protein levels were strongly upregulated following infection with the POR1 and POR2 strains but remained close to baseline following infection with POR3 (Fig. 5B).

### Mucosal invasion and tissue pathology depend on the T3SS2 effector VopC.

**FIG 6:**
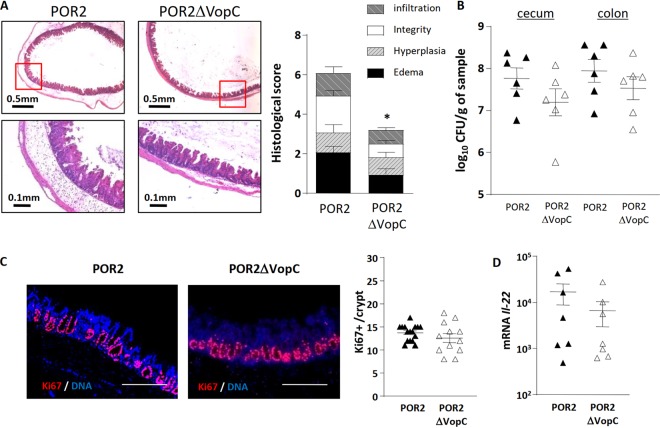
Mucosal invasion and tissue pathology depend on the T3SS2 effector VopC. (A) Representative H&E staining images of the ceca of germfree mice infected with the POR2 or the POR2*ΔvopC* mutant strain at 21 h p.i. as well as the tissue pathology scores. (B) Pathogen burdens (CFU) in the ceca and colons are similar between strains. (C) Cecal crypt IEC proliferation was determined by Ki-67 (red) staining and counting the number of Ki-67-positive cells per crypt. The nuclei were stained with DAPI (blue). Bars, 200 μm. (D) Cecal levels of *Il-22* mRNA were similar between POR2- and POR2*ΔvopC*-infected mice. *, *P* < 0.05, by Student’s *t* test.

The above studies demonstrate that the gastroenteritis seen during V. parahaemolyticus infection is largely dependent on the T3SS2. Previous *in vitro* studies have identified the T3SS2 effector VopC as critical to the ability of V. parahaemolyticus to invade IECs *in vitro* (32). While VopC was shown to be dispensable in the rabbit infant and ileal loop models (33), the prominent tissue invasion seen in infected germfree mice led us to compare the POR2 strain with a POR2 strain specifically lacking VopC (POR2*ΔvopC*). At 21 h p.i., mice were euthanized, and cecal tissues were removed and histologically stained. As expected, infection with the POR2 strain caused severe tissue pathology and IEC sloughing, along with crypt hyperplasia (Fig. 6A). In contrast, this damage was significantly attenuated in mice infected with the POR2*ΔvopC* strain (Fig. 6A). While the gastroenteritis was not as attenuated as we observed in POR3-infected mice (Fig. 4B), compared to the POR2 strain, tissue pathology scores obtained in a blind manner revealed the POR2*ΔvopC* mutant caused significantly less inflammation and edema and essentially no IEC damage or sloughing, although there was still evidence of crypt hyperplasia (Fig. 6A). While we attempted to show complementation of VopC *in vivo*, such results were unsuccessful, similar to what has been previously seen with Vibrio cholerae wherein the lack of complementation may be due to polar effects (47). Originally, we tried using a plasmid-based complementation that was unsuccessful. Since we hypothesized that this could be due to the plasmid-based VopC being under the control of a foreign promoter, which often alters the level and/or timing of the effector gene expression, we created a chromosome-based complement, which involved replacing the entire VopC gene into its original location. We did, however, include a tag to identify VopC, which may be contributing to the difficulties in achieving full complementation. To confirm that the deletion of VopC was the cause for the absence of observed invasion, we used a VopC complemented strain in an *in vitro* invasion assay and observed invasion, albeit at a lower efficiency than with the POR2 strain (Fig. S4). In addition, we demonstrated that secretion of VopC is rescued in the VopC complemented strain but also at a lower efficiency than in the POR2 strain (Fig. S5). Sequencing of the POR2, *ΔvopC*, and *ΔvopC*::*vopC Flag* strains revealed a mutation in both the *ΔvopC* and *ΔvopC*::*vopC Flag* strains that carried a G40C transition, which causes an A14P amino acid change in a *lacI* family transcriptional regulator. LacI homologues have been implicated in carbohydrate metabolism, and deletion of *lacI* is correlated with gas vesicle abnormalities in the plant pathogen *Serratia* (48, 49). Although unlikely, the single amino acid change seen in the sequencing could also contribute to the lack of complementation in the *in vivo* assays. Cumulatively, we believe that lower efficiency of complementation in the *in vitro* secretion and invasion assays may be contributing to the lack of complementation in the *in vivo* assays. The fact that we do see complementation *in vitro* supports the molecular Koch postulate and therefore the premise that lack of invasion in the ΔVopC strain is caused by the deletion of the VopC effector.

10.1128/mBio.02608-19.4 FIG S4 *In vitro* invasion assay of HeLa cells infected with different strains of V. parahaemolyticus. HeLa cells were infected with POR2, POR2Δ*vopC*, or POR2Δ*vopC*::VopC for 1.5, 3, 4.5, and 6 hours after gentamicin treatment. Host cell lysates were serially diluted and plated onto MMM agar plates for intracellular bacterial colony counting (CFU/ml). Numbers are expressed as an average from three technical replicates for one of three independent experiments. Error bars represent standard deviation from the mean. Asterisks represent statistical significance (*, *P* < 0.05; ***, *P* < 0.001; ***, *P* < 0.0001) determined using Student’s *t* test. Download FIG S4, PDF file, 0.2 MB. Copyright © 2019 Yang et al. 2019 Yang et al. This content is distributed under the terms of the Creative Commons Attribution 4.0 International license.

10.1128/mBio.02608-19.5 FIG S5 Murine model of *V. parahaemolyticus*. Download FIG S5, TIF file, 0.09 MB. Copyright © 2019 Yang et al. 2019 Yang et al. This content is distributed under the terms of the Creative Commons Attribution 4.0 International license.

**FIG 7:**
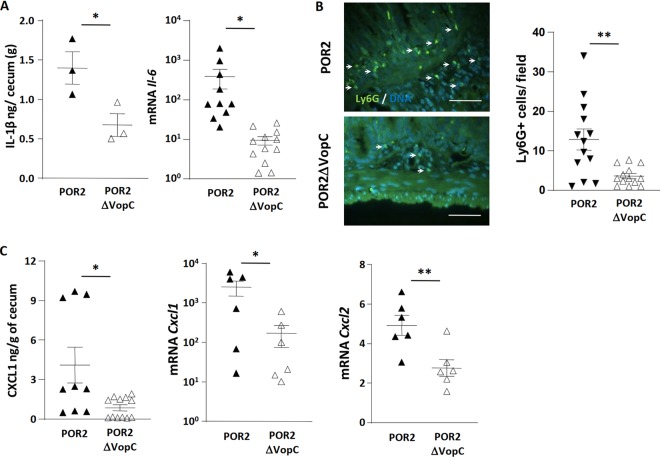
Proinflammatory cytokine production and neutrophil recruitment are driven by T3SS2 effector VopC. (A) The expression of proinflammatory cytokines (IL-1β and *Il-6*) was determined from cecal tissues. (B) Ly6G-positive neutrophil (green) recruitment (white arrows) was demonstrated by immunostaining and counting Ly6G-positive cells/high-power field. Representative images were shown. Nuclei were stained with DAPI in blue. Bars, 50 μm. (C) The expression of the neutrophil chemokines CXCL1 (protein and mRNA) and *Cxcl2* (mRNA) was measured from infected ceca. In the graphs, bars show the mean ± SEM, and each symbol represents an individual mouse from 2 to 3 independent experiments. *, *P* < 0.05; **, *P* < 0.01, by two-tailed Student’s *t* test.

To define the basis for this reduced pathology, we measured pathogen burdens, recovering comparable numbers of V. parahaemolyticus bacteria from the ceca of POR2*ΔvopC-* and POR2-infected mice (Fig. 6B). When we assessed IEC proliferation, we noted Ki-67-positive staining levels were similar between the two groups, as was transcription of the *Il-22* gene (Fig. 6C and D). In keeping with the attenuated tissue damage, the typical infection-induced increases in levels of mature IL-1β, as well as transcription of the proinflammatory cytokine *Il-6* (Fig. 7A) and infiltration of Ly6G-positive neutrophils into the infected cecal mucosa, were significantly attenuated in POR2*ΔvopC* mice (Fig. 7B). Corresponding with the reduced neutrophil infiltration, levels of the neutrophil chemokines *Cxcl1* and *Cxcl2* (Fig. 7C) were significantly attenuated in tissues infected with the POR2*ΔvopC* strain.

**FIG 8:**
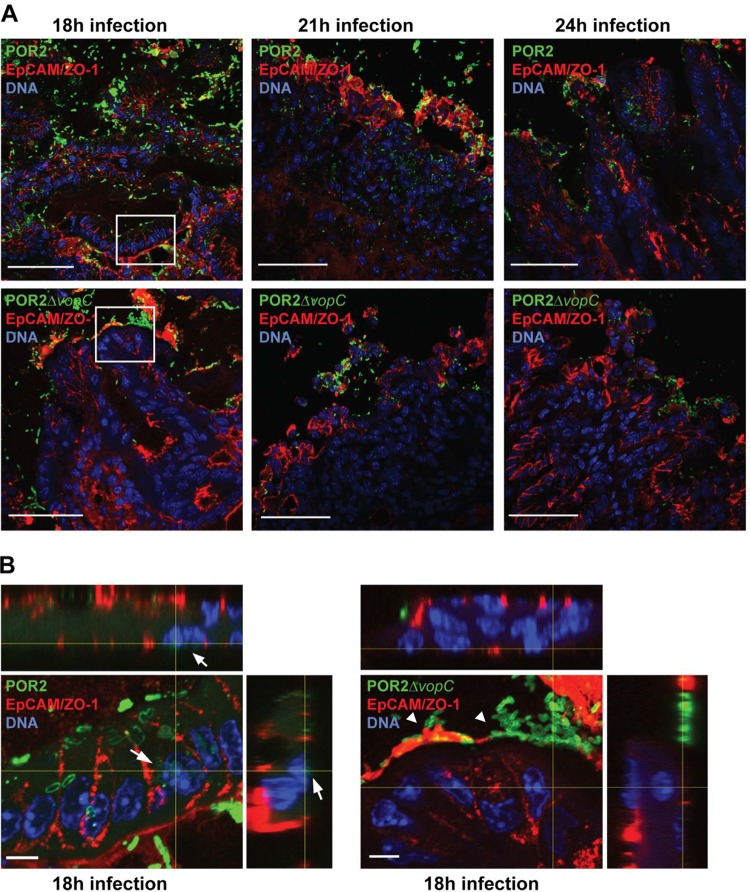
POR2, but not the POR2*ΔvopC* mutant strain, invades the cecal epithelia; however, both strains cause epithelial damage in infected germfree mice. (A) Ceca of germfree mice infected with either POR2 or POR2Δ*vopC* for 18, 21, and 24 h. Ceca were collected and processed for immunofluorescent staining for V. parahaemolyticus (green), cell membrane was stained with anti-EpCAM and anti-ZO-1 (red), and DNA was stained with Hoechst stain (blue). Bars, 50 μm. Boxed areas are magnified in panel B. (B) Orthogonal projections of stacks of confocal micrographs of germfree mouse ceca infected with either POR2 or POR2Δ*vopC* for 18 h. Bars, 5 μm. White arrows indicate invading bacteria in close proximity to the cell nucleus. White arrowheads indicate adherent bacteria on the epithelial surface.

To better define the actions of VopC *in vivo*, we used immunofluorescent staining to localize V. parahaemolyticus within the cecal tissues of mice infected with either POR2 or the POR2*ΔvopC* strain. *In vitro* studies have shown that V. parahaemolyticus invades, propagates within, and lyses host cells within a few hours (50–52), much faster than other intracellular pathogens such as *Salmonella* spp. (53). Considering this, and the severe tissue damage demonstrated in the POR2-infected mice at 21 h p.i., we collected tissues 18 h p.i., when infection was beginning and tissue damage was still limited, as well as at 21 and 24 h p.i., when damage was overt. At 18 h p.i., we observed clear invasion of the cecal mucosa of mice infected with POR2 but not POR2*ΔvopC* (Fig. 8A and B). Moreover, the POR2 strain appeared to invade epithelial cells as evidenced through z-stacks of images in tissue slices where the POR2 bacteria were adjacent to nuclei (Fig. 8B). In contrast, the POR2*ΔvopC* bacteria remained luminal, or adherent to the epithelial surface, with few signs of tissue invasion (Fig. 8B). Similar findings, albeit with more tissue pathology, were observed at 21 and 24 h p.i. These findings confirm that the T3SS effector VopC plays an important role in V. parahaemolyticus’ interactions with its host and suggest that *in vivo* it plays a crucial role in the ability of this pathogen to invade the cecal mucosa and cause gastroenteritis.

## DISCUSSION

We describe a novel and robust model of V. parahaemolyticus-induced gastroenteritis, using adult germfree mice. While significant progress has been made characterizing the virulence mechanisms used by V. parahaemolyticus to infect IECs *in vitro*, the limited options for studying how V. parahaemolyticus infects its mammalian hosts have hampered our understanding of its pathogenesis. Compared to other animal species, the array of genetic and immunological tools available for mice makes this species the preferred choice for *in vivo* modeling. By infecting germfree mice, we were able to define the individual contributions of the virulence systems expressed by V. parahaemolyticus. We also observed that the resident microbiota precluded V. parahaemolyticus colonization of the intestine of conventionally raised mice, whereas germfree mice allowed us to bypass this microbial competition. Although the POR3 strain of V. parahaemolyticus (lacking the T3SS2) was able to colonize the intestinal lumen of these mice, we confirmed studies in the rabbit ileal loop model that this strain is largely avirulent, as it was unable to infect the intestinal mucosa. Our findings differ, however, from the rabbit model of V. parahaemolyticus infection, in that the T3SS2 controls not only V. parahaemolyticus’ ability to adhere to IECs but also their ability to invade the intestinal mucosa.

Infection with the V. parahaemolyticus clinical isolate RIMD resulted in severe gastroenteritis characterized by submucosal edema, crypt ulceration and hyperplasia, IEC damage, and infiltration by neutrophils. Similar signs of inflammation were present in the ceca of mice infected with the POR1 and POR2 strains but not with POR3. From these phenotypes, we infer that a functional T3SS2 contributes to V. parahaemolyticus-induced intestinal damage. The lack of gastroenteritis in those mice infected with POR3 suggests the T3SS1 does not on its own contribute to the pathogenic effects of V. parahaemolyticus. The inflammation observed in animals infected with the POR2 strain likely involved the translocation of bacterial effectors that are known to disrupt the actin cytoskeleton, compromising the integrity of the polarized epithelium and allowing bacteria to invade the underlying mucosal tissues (14).

Based on the key role played by the T3SS2 in this model, we tested the potential role of the effector VopC in infection. Previous *in vitro* studies have identified a number of virulence properties mediated by VopC, including the invasion of IECs, but curiously this effector appeared to have no effect in the rabbit ileal loop model (33). By testing a strain lacking VopC, on a POR2 background, we found that VopC plays a critical role in tissue invasion in germfree mice. While the POR2 strain was found to heavily adhere to the mucosal surface, causing IEC sloughing as well as invading and spreading through the underlying lamina propria, only the mucosal adherence occurred with the POR2*ΔvopC* strain. The lack of tissue invasion by this strain appeared to dramatically reduce the severity of the gastroenteritis. However, much like other noninvasive pathogens such as Citrobacter rodentium (54), the POR2*ΔvopC* strain still caused significant crypt hyperplasia and IL-22 production.

These findings suggest that the VopC effector plays an important role in the *in vivo* pathogenesis of V. parahaemolyticus. It is notable that tissue invasion is not seen in the rabbit ileal loop model, and thus, in the absence of this phenotype, VopC was not found to impact *in vivo* virulence (33). While it is intriguing that such differences in virulence strategies are seen between models, they may reflect disparities in the pathogen’s ability to interact with mouse versus rabbit intestinal mucosa or differences in the sites of infection (cecum versus ileum). Alternatively, the timing of the infection may be critical for dissecting the actions of different virulence factors. For example, S. enterica serovar Typhimurium causes severe enterocolitis in mice by 24 h p.i., but it was only when cecal tissues were assessed at earlier time points (12 to 18 h p.i.) that intracellular *Salmonella* bacteria were identified within infected IECs (55). Notably, when studied *in vitro*, V. parahaemolyticus infects IECs, intracellularly replicates, and eventually lyses the cell, all within 6 h (13). For the rabbit ileal loop model, infected tissues are always observed at 24 h p.i. in order to assess maximal tissue damage, whereas the infected tissues of infant rabbits are examined at 38 h p.i. (10, 33). These later time points may explain why tissue invasion is seen in the germfree mouse model (18 to 21 h p.i.) compared to the rabbit models. These findings highlight the value of testing particular virulence factors in multiple model systems, since each model has its strengths and weaknesses.

Consistent with previous observations (10, 56, 57), the expression of several neutrophil chemokines, cytokines, and antimicrobial factors was significantly increased over baseline in RIMD-, POR1-, and POR2-infected mice at 21 h. CXCL1 and CXCL2 recruit neutrophils and other leukocytes to sites of infection and inflammation. Similarly, the antimicrobial factor Reg3γ was increased during infection, as was the multifunctional cytokine IL-22. While these host responses are likely focused on creating an antimicrobial environment to limit the growth and spread of the invading pathogen, they also contribute to the gastroenteritis that develops in this model. Future studies infecting gnotobiotic mouse strains deficient in host cytokines or injecting them with neutralizing antibodies will help define their specific roles and whether their actions prove ultimately beneficial or harmful during infection.

While there are concerns that the sterility of germfree mice may provide unrealistic susceptibility to microbes, our findings were reassuring, since we found that infection with the POR3 strain of V. parahaemolyticus, which lacks the virulence factors thought to contribute to enteropathogenesis, did not elicit a notable inflammatory response. Infection with POR3 may therefore be considered a V. parahaemolyticus negative control for enteropathogenicity in this model and indirectly affirms the results obtained with the other, enteropathogenic strains of V. parahaemolyticus. Moreover, despite the heavy pathogen burdens and severe intestinal damage seen in infected mice, translocation out of the gut was only rarely seen. These findings support our previous studies using the pathogen C. rodentium to infect germfree mice, showing that relevant virulence pathways and host defense responses can be studied in germfree mice upon infection.

In summary, establishing a germfree mouse model of V. parahaemolyticus infection presents a useful tool for the further study of this pathogen. The gastroenteritis demonstrated in this model is characterized by submucosal edema, crypt ulceration and hyperplasia, epithelial damage, and neutrophil infiltration; these phenotypes are also characteristic of intestinal tissues isolated from humans infected with V. parahaemolyticus. Consistent with previous studies (8, 11), the T3SS1 is not required for enteric pathogenesis, whereas the T3SS2 is a necessary requirement for V. parahaemolyticus to cause gastroenteritis. Many future questions regarding the virulence mechanisms of V. parahaemolyticus can be addressed using this system. For example, we have already used this model to define the role of the effector VopC in the *in vivo* pathogenesis of V. parahaemolyticus. Thus, infecting germfree mice should prove valuable in elucidating the effects of additional effectors of V. parahaemolyticus through the use of additional bacterial mutant strains. The study of the mechanistic contributions of individual effectors may come full circle by elucidating the relative contributions of T3SS1 and T3SS2 in this acute animal model of V. parahaemolyticus infection.

## MATERIALS AND METHODS

### Bacterial strains and culture.

The strains used include the V. parahaemolyticus clinical isolate RIMD2210633 (serotype O3:K6) and its derivatives POR1, POR2, and POR3, all provided by T. Honda from Osaka University, Japan. POR1 contains an in-frame deletion for *tdhAS*, which encodes the two TDH hemolysins (8). POR2 and POR3 are derivatives of POR1, each containing in-frame deletions for *vscN1* (T3SS1 deficient) and *vscN2* (T3SS2 deficient), respectively (8). POR2`ι>ΔvopC`/ι> was generated as described previously (32). To culture V. parahaemolyticus, strains were streaked onto modified Luria-Bertani (MLB) agar plates (1% tryptone, 0.5% yeast extract, 3% NaCl, and 1.5% Bacto agar per liter) and incubated at 30°C overnight. Single bacterial colonies were cultured overnight at 30°C in MLB medium. Prior to gavage, overnight cultures were diluted to an OD_600_ of 0.5 in MLB medium and incubated at 37°C with shaking for 90 min. Cultures were centrifuged and resuspended in sterile phosphate-buffered saline (PBS) at a concentration of 2 × 10^10^ CFU/ml.

For reconstitution of POR2*ΔvopC*, three amplicons consisting of a 1-kb upstream region, full-length VopC with a C-terminal Flag tag, and a 1-kb downstream region of VopC were amplified with the primer sets CATAAGATCTAGCCCAACAGATGGCTCGCA and TCTGTTAATAGCAAATTAGTGCTATTGAAAAATGGTGACTACAAAGACGATGACGACAAGTAATCATAAATGCAACGTATATTTCTTTAG, TTACTTGTCGTCATCGTCTTTGTAGTCACCATTTTTCAATAGCACTAATT and ATGCCAATATTAAATATTAGTA, and TCTGTATTACTAAATTTACTAATATTTAATATTGGCATATGGACCTCACTATTCTAAATTAAAAACC and ATCGGGGCCCATTCTTAATAAGTCAGGAGGG, respectively, and fused together using overlap extension PCR. This fused construct was cloned into pDM4, a Cm^r^ Ori6RK suicide plasmid between the restriction sites BglII and ApaI. The resulting construct was inserted into POR2*ΔvopC* via conjugation by S17-1 (λ*pir*) Escherichia coli. Transconjugants were selected for on minimal marine medium (MMM) agar containing 25 μg/ml chloramphenicol. Subsequent bacterial growth on MMM agar containing 15% sucrose (wt/vol) allowed for counterselection and curing of *sacB*-containing pDM4. Complementation was confirmed by PCR and sequencing analysis.

### *In vitro* secretion assay.

For the secretion of T3SS2 effector VopC, V. parahaemolyticus strains were grown overnight in MLB at 30°C, diluted to an OD_600_ of 0.3 in LB supplemented with 0.05% bile salts, and grown at 37°C for 3 h as previously described (58). To check for the expression of VopC, bacterial cultures with an OD_600_ of 0.5 were pelleted and resuspended in 2× Laemmli buffer. For the secretion, bacterial culture supernatants were filtered with an 0.22-μm filter, precipitated with deoxycholate (150 μg/ml) and trichloroacetic acid (7% [vol/vol]) for 16 h at 4°C and centrifuged at 16,000 × *g* for 20 min to pellet precipitated proteins, washed twice with acetone, and then resuspended in 2× Laemmli buffer. Immunoblotting was carried out to check for VopC expression and secretion, and Coomassie blue staining was used to assess the total protein load.

### *In vitro* invasion assay.

HeLa cells were plated in triplicate in a 24-well tissue culture plate at 7 × 10^4^ cells per well and grown for 16 to 18 h. Overnight-grown bacterial cultures in MLB at 30°C were diluted to an OD_600_ of 0.3 in MLB supplemented with 0.05% bile salts and grown at 37°C for 90 min to induce T3SS2. Induced V. parahaemolyticus cultures were then used to infect HeLa cells at an MOI of 10. All infections were carried out at an MOI of 10. Gentamicin was added at 100 μg/ml to each well after 2 h of infection to kill extracellular bacteria. At each indicated time point, monolayers of HeLa cells were washed with PBS, and cells were lysed by incubation with PBS containing 0.5% Triton X-100 for 10 min at room temperature with agitation. Serial dilutions of lysates were plated on MMM plates and incubated at 30°C overnight for subsequent CFU enumeration.

### Mouse treatments and infection.

All studies were performed according to protocols approved by the University of British Columbia. Using a 1-ml syringe attached to a blunt-tipped metal gavage needle, both male and female C57BL/6 mice between 8 and 12 weeks of age were orally gavaged with 2 × 10^9^ CFU of V. parahaemolyticus (see above) in a volume of 100 μl sterile PBS or with 100 μl of PBS alone (control). Infected mice were housed in cages maintained in a sterile hood during the course of infection and were euthanized 21 to 72 h following infection by inhalation of 4% isoflurane, followed by cervical dislocation. The weight of each mouse was recorded both prior to infection and upon euthanization.

### Tissue dissection and sample collection.

Upon euthanization, mice were dissected using autoclave-sterilized instruments. The cecum and colon were removed and placed in a sterile petri dish. Small sections (0.5 cm) of tissue were then placed into separate, sterile tubes containing either formalin fixative or RNAlater for RNA isolation. Tissue samples for RNA isolation were stored at −80°C. The remaining tissues were slit longitudinally for collection of luminal contents, which were resuspended in 1 ml sterile PBS in a sterile microcentrifuge tube and placed on ice prior to dilution plating (see below). Instruments were resterilized, and the remaining tissues were vigorously washed in PBS three to five times to remove residues and placed in 1 ml sterile PBS for further analysis. Mesenteric lymph nodes (MLN), spleen, and liver were extracted to measure their bacterial loads.

### Bacterial dilution plating and enumeration.

Luminal contents from the colon, cecum, and colon were placed in 2 ml U-bottom tubes containing 1 ml of sterile PBS and steel beads (Qiagen). All samples were weighed and subsequently homogenized with a MixerMill 301 homogenizer (Retsch, Newtown, PA). Six successive serial dilutions in PBS yielded 10^−1^ to 10^−6^ dilutions. Ten microliters of each dilution was plated in triplicate onto MLB agar plates. Plates were incubated at 30°C overnight. The spleen, liver, and MLN were also collected from each animal and then homogenized and plated as outlined above. Plates were incubated at 30°C overnight.

Following incubation, bacteria were enumerated by counting the number of colonies per plate. The number of CFU per weight of the sample was calculated by multiplying the number of colonies by the dilution factor of the plate. Data were compiled in Microsoft Excel, and graphs were generated using Prism 7 (GraphPad).

### Slide preparation and hematoxylin and eosin staining.

Fixed tissue samples were submitted to the Histology Core Facility at British Columbia Children’s Hospital Research Institute for paraffin processing, embedding, and sectioning following established protocols. H&E staining of paraffin-embedded tissue sections was performed by the Histology Core Facility at British Columbia Children’s Hospital Research Institute with commercially prepared solutions and following established protocols.

### qPCR analysis.

Tissue (5 mm) was removed from the middle of the cecum, transferred into RNAlater buffer (Qiagen), and stored at −80°C. Total RNA was extracted using a Qiagen RNeasy kit according to the manufacturer’s protocol. In brief, the RNA-stabilized tissues were homogenized with a MixerMill 301 homogenizer (Retsch, Newtown, PA) in the lysis buffer of the kit. Total RNA was quantified using a NanoDrop spectrophotometer (ND1000). cDNA was constructed using the Omniscript reverse transcription kit (Qiagen) according to the manufacturer’s instructions. The cDNA was added to a PCR mixture (10 μl of Bio-Rad SYBR green supermix; primers were at a final concentration of 300 nM; final reaction volume, 20 μl). Quantitative PCR (qPCR) was carried out using a Bio-Rad Opticon system. The primers used for qPCR are listed in Table S2 in the supplemental material, and their efficiencies were in the range of 0.9 to 1.1. Quantification was determined using the comparative cycle threshold (*C_T_*) method, relative to housekeeping gene *Gapdh*. The average of baseline obtained from uninfected control germfree mice was normalized to 1 for relative expression.

10.1128/mBio.02608-19.6 TABLE S2 List of primers for qPCR analysis. Download Table S2, DOCX file, 0.01 MB. Copyright © 2019 Yang et al. 2019 Yang et al. This content is distributed under the terms of the Creative Commons Attribution 4.0 International license.

### Immunofluorescence staining.

Slides were deparaffinized in 2 successive 5-min washes in xylene, followed by a series of 3-min washes in graded ethanol (100%, 100%, 95%, 70%) and a 10-min rinse under running tap water. Antigen retrieval was performed by incubation in fresh buffer containing 10 mM sodium citrate and 0.05% Tween 20, pH 6.0, at 98°C for 20 min. Tissues were permeabilized with 1% Triton X-100 and 0.05% Tween 20 for 10 min at room temperature. Following a 5-min wash in 1× PBS, tissue blocking was done in the presence of PBS-T (0.1% Triton X-100, 0.05% Tween 20 in PBS) with 2% BSA for 30 min at room temperature, washed in 1× PBS for 5 min. Slides were incubated with rabbit polyclonal anti-Ki-67 primary antibody (Abcam), diluted 1:1,000 in blocking buffer or anti-V. parahaemolyticus O3 antibody (Abcam, ab78751), at 1:50 dilution in PBS-T with 1% BSA, overnight at 4°C in a humid chamber. Slides were subsequently washed in three successive 5-min washes in TBS-T and incubated with goat anti-rabbit Alexa Fluor 568-conjugated secondary antibody (Life Technologies) diluted 1:500 in blocking buffer 1 for 30 min at room temperature. Host cells were stained with anti-EpCAM (Santa Cruz; sc-53532) and anti-ZO-1 (Life Technologies; 339100) antibodies, both at 1:50 dilution in PBS-T with 1% BSA, and incubated for 1 h at room temperature. Slides were washed 3 times successively in 1× TBS-T in a light-protected chamber. To stain nuclei, slides were incubated with Hoechst stain (Sigma) diluted 1:5,000 in blocking buffer A for 15 min in the dark at room temperature. Slides were washed once in 1× TBS-T for 5 min. Stained tissues were mounted using ProLong Gold antifade (Molecular Probes) containing 4′,6-diamidino-2-phenylindole (DAPI), a DNA-staining dye, or with Hoechst 33342 (ThermoFisher, H3570). Images were captured using a Zeiss AxioImager microscope equipped with an AxioCamHRm camera operating through AxioVision software (version 4.4).

### Histology and scoring.

Histopathological scores were determined using H&E-stained tissues by 2 independent observers blinded to the identity of samples. Tissue damage was assessed for submucosal edema (0, no edema; 1, mild (<50% of the diameter of the entire intestinal wall [tunica muscularis to epithelium]; 2, moderate [50 to 80%]; 3, profound edema), epithelial hyperplasia (scored based on the percent change in crypt height compared to that of the control crypts; 0, no change; 1, 1% to 50% change; 2, 51% to 100%; 3, >100%), inflammatory cell infiltration (0, none; 3, severe), and epithelial integrity (0, no pathological changes detectable; 1, epithelial desquamation [a few cells sloughed, surface rippled]; 2, erosion of epithelial surface [epithelial surface rippled, damaged]; 3, epithelial surface severely disrupted/damaged, large amounts of cell sloughing). The maximum possible score was 12.

### Cytokine measurements.

The ceca of uninfected and infected (21-h) mice were removed, slit longitudinally, and washed three times in PBS to remove the luminal contents. Tissue was transferred into 2 ml U-bottom tubes containing 1 ml of PBS and steel beads (Qiagen). The samples were weighed and subsequently homogenized with a MixerMill 301 homogenizer (Retsch, Newtown, PA). The homogenates were centrifuged at 12,000 × *g* for 10 min at 4°C, and the collected supernatant was stored at −80°C for later analysis. IL-1β and IFN-γ were quantified using commercially available ELISA kits (MBL and R&D Systems) according to the manufacturer’s protocols.

### TUNEL staining.

TUNEL staining was performed using the Dead End fluorometric TUNEL system (Promega) according to manufacturer’s instructions. Briefly, slides were deparaffinized, and V. parahaemolyticus staining was performed using anti-V. parahaemolyticus O3 antibody as described above. Sections were then incubated with equilibration buffer for 10 min at room temperature followed by incubation with rTdT nucleotide mix at 37°C for 1 h, and nuclei were stained with Hoechst stain. All imaging was performed on a Zeiss LSM 800 confocal microscope, and images were converted using ImageJ (NIH).

### Sequencing of *Vibrio* strains.

Whole-genome sequencing for the strains POR2, POR2*ΔvopC*, and complemented POR2*ΔvopC*::*vopC Flag* was carried out using the Nextera XT kit (Illumina), followed by a single-end 150-cycle, high-output sequencing on the NextSeq (Illumina) at the Tufts University core facility (www.tucf.com). The resulting 39 to 56 million reads, with a coverage of >1,100, were used for a *de novo* genome assembly on the POR2 reads using CLC Genomics Workbench software (v11) (Qiagen). This yielded 95 contigs greater than 300 bp in size, for a total genome size of 5,083,670 bp. The reads from the VopC deletion and complemented strain were then mapped to the POR2 contigs. The mapping files were then put through the Basic Variant Detection algorithm in CLC Genomics Workbench.

### Statistical analysis.

The results were analyzed using GraphPad Prism 8. A normality test was run for each data set to determine if it displayed a normal distribution. Statistical significances were calculated using the two-tailed Student *t* test or Mann-Whitney test as indicated. Results presented are expressed as the mean value ± standard error of the mean (SEM) unless otherwise stated. Asterisks represent statistical significance: *, *P* < 0.05; **, *P* < 0.01; ***, *P* < 0.001; ****, *P* < 0.0001.

### Data availability.

The sequencing files are deposited in the NCBI GenBank database under accession numbers SAMN13181012, SAMN13181013, and SAMN13181014.
