# Supplementary figures and images for: A Novel Mouse Model of Enteric Vibrio parahaemolyticus Infection Reveals that the Type III Secretion System 2 Effector VopC Plays a Key Role in Tissue Invasion and Gastroenteritis
Source: mBio. 2019 Dec 17;10(6):e02608-19. doi: 10.1128/mBio.02608-19 (PMC6918077; doi:10.1128/mBio.02608-19)

# Supplemental Figure 1

# Murine model of *V. parahaemolyticus* (HY, MS et. al.)

S1A

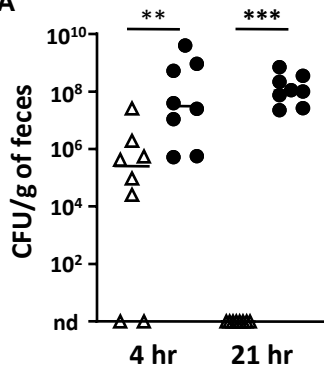

S1B

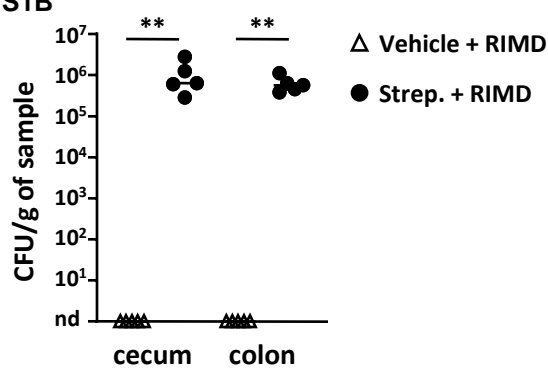

S1C

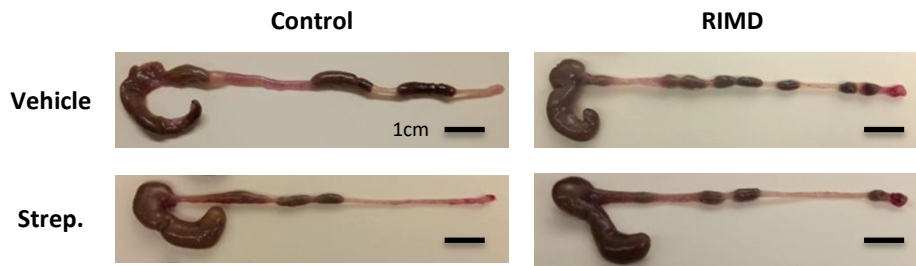

S1D

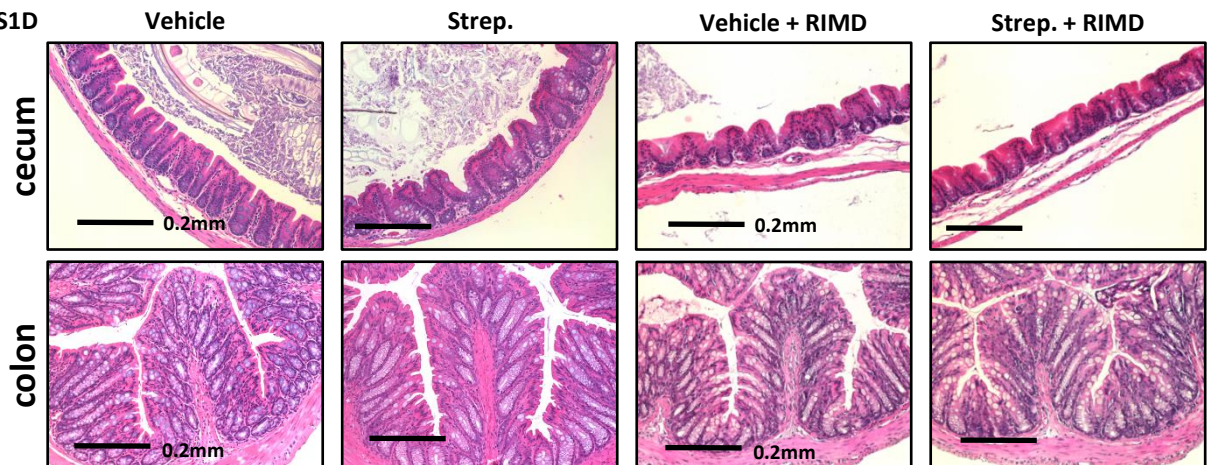

S1E

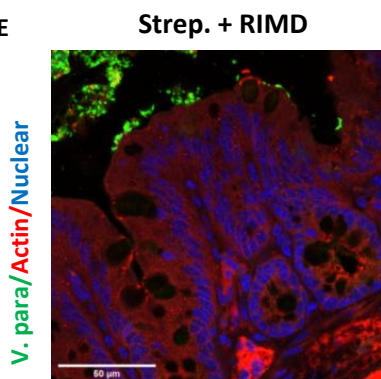

Supplement: FIG S1 [file mBio.02608-19-sf001.pdf]

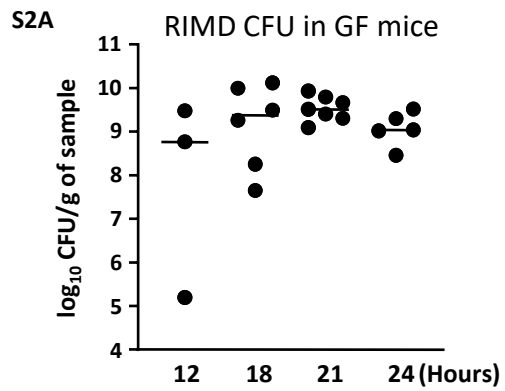

S2B

Control

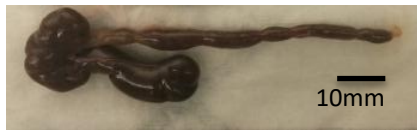

RIMD

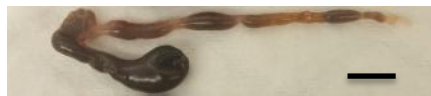

S2C

Control

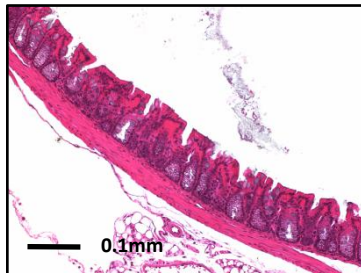

RIMD

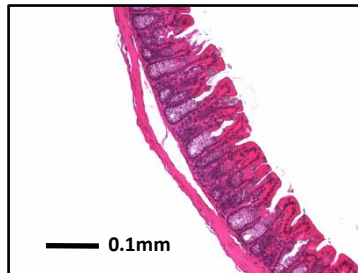

Supplement: FIG S2 [file mBio.02608-19-sf002.pdf]

Supplemental Figure 3 Murine model of *V. parahaemolyticus* (HY, MS et. al.)

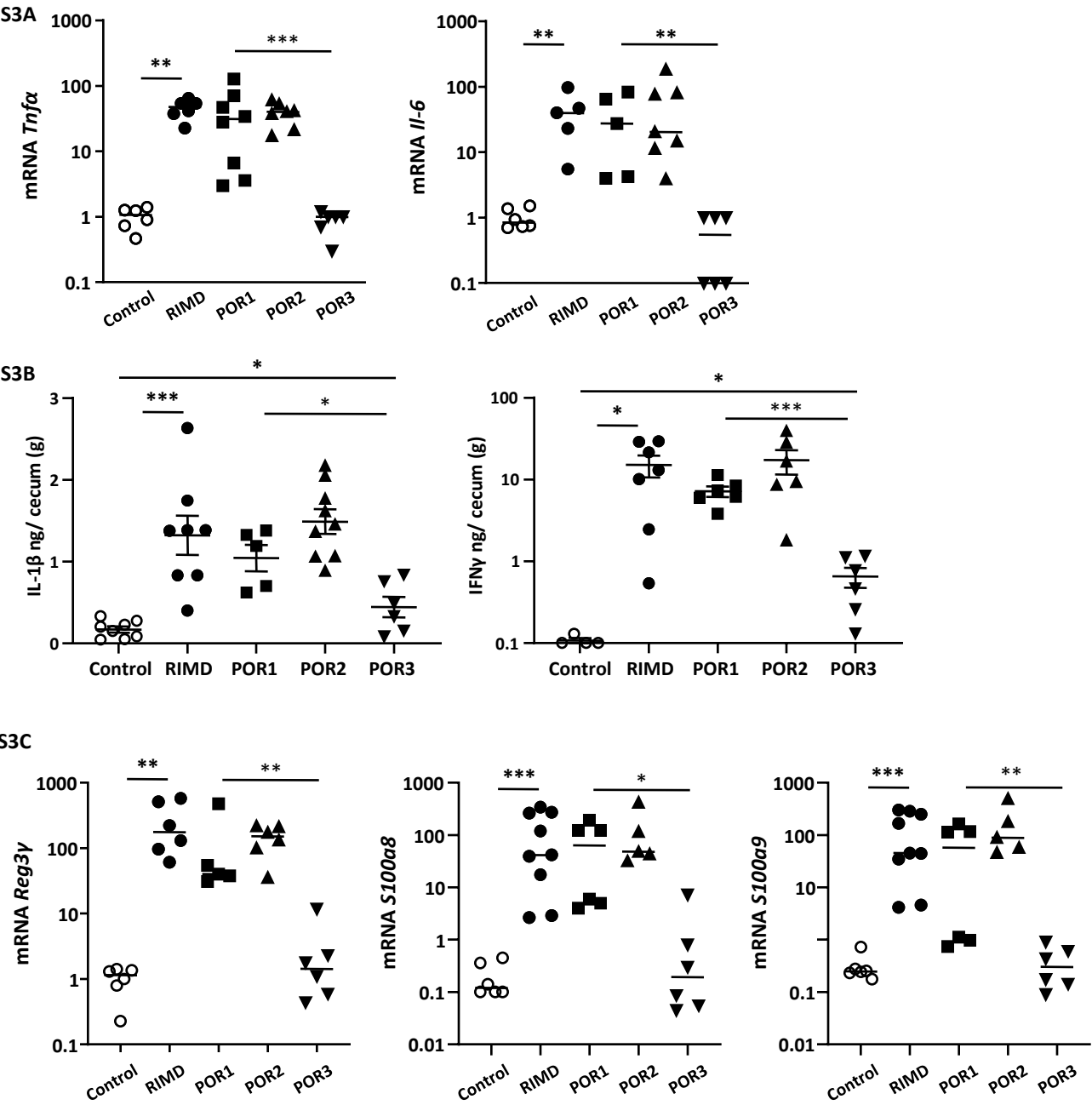

Supplement: FIG S3 [file mBio.02608-19-sf003.pdf]

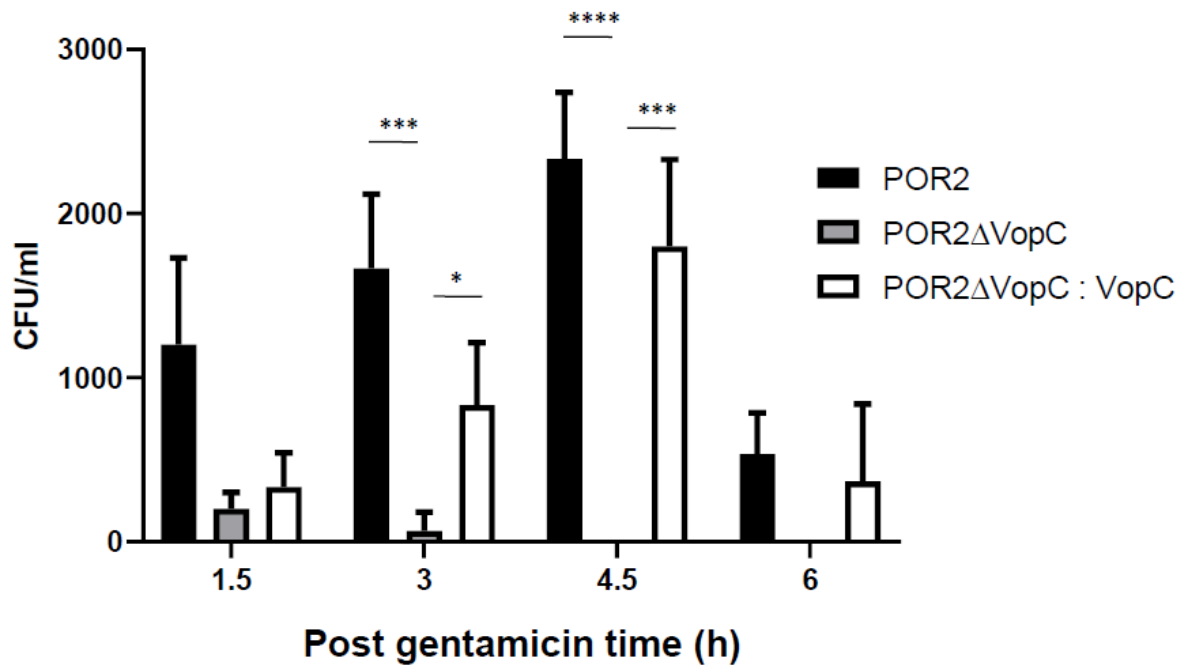

Supplement: FIG S4 [file mBio.02608-19-sf004.pdf]

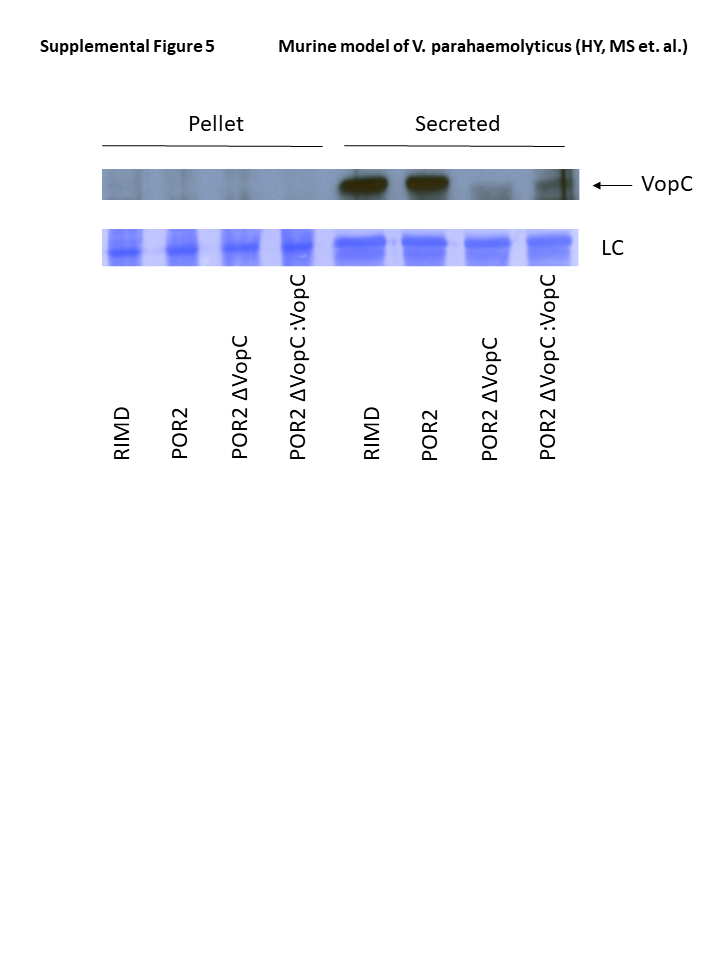

Supplement: FIG S5 [file mBio.02608-19-sf005.tif]
