# Supplementary material for: A Novel Mouse Model of Enteric Vibrio parahaemolyticus Infection Reveals that the Type III Secretion System 2 Effector VopC Plays a Key Role in Tissue Invasion and Gastroenteritis
Source: mBio. 2019 Dec 17;10(6):e02608-19. doi: 10.1128/mBio.02608-19 (PMC6918077; doi:10.1128/mBio.02608-19)
Supplement: TABLE S1 [file mBio.02608-19-st001.pdf]

Table 1. RIMD2210633 and its mutant strains

Strain	TdhA & TdhS	T3SS1	T3SS2	effector VopC
RIMD	+	+	+	+
POR1	-	+	+	+
POR2	-	-	+	+
POR3	-	+	-	-
POR2 ΔVopC	-	-	+	-
