# Supplementary material for: A Novel Mouse Model of Enteric Vibrio parahaemolyticus Infection Reveals that the Type III Secretion System 2 Effector VopC Plays a Key Role in Tissue Invasion and Gastroenteritis
Source: mBio. 2019 Dec 17;10(6):e02608-19. doi: 10.1128/mBio.02608-19 (PMC6918077; doi:10.1128/mBio.02608-19)
Supplement: TABLE S2 [file mBio.02608-19-st002.docx]

| Gene | Forward (5' to 3') | Reverse (5' to 3') |
| --- | --- | --- |
| *Gapdh* | ATG ACC TTG CC ACA GCC | CCC ATC ACC ATC TTC CAG |
| *Reg3γ* | TGC CTA TGG CTC CTA TTG CT | CAC TCC CAT CCA CCT CTG TT |
| *Tnfα* | CAT CTT CTC AAA ATT CGA GTG ACA A | TGG GAG TAG ACA AGG TAC AAC CC |
| *Il-6* | GAG GAT ACC ACT CCC AAC AGA CC | AAG TGC ATC ATC GTT GTT CAT |
| *Il-22* | ACC TTT CCT GAC CAA ACT CA | AGC TTC TTC TCG CTC AGA CG |
| *Cxcl1* | TGC ACC CAA ACC GAA GTC AT | TTG TCA GAA GCC AGC GTT CAC |
| *Cxcl2* | CCT GCC AAG GGT TGA CTT CA | TTC TGT CTG GGC GCA GTG |
| *S100a8* | GCC GTC TGA ACT GGA GAA GGC CT | TCA CCA TCG CAA GGA ACT CCT CG |
| *S100a9* | AGG AAG GAC ACC CTG ACA CCC | ACG TGG GTT GTT CTC ATG CAG C |

Table 2 qPCR primer list
